# Dravet Syndrome in Lebanon: First Report on Cases with *SCN1A* Mutations

**DOI:** 10.1155/2019/5270503

**Published:** 2019-01-21

**Authors:** Saada Alame, Eliane El-Houwayek, Caroline Nava, Sandra Sabbagh, Ali Fawaz, Anne-Celine Gillart, Dana Hasbini, Christel Depienne, André Mégarbané

**Affiliations:** ^1^Neuropediatrics Department, Lebanese University, Beirut, Lebanon; ^2^Pediatrics, Lebanese University, Beirut, Lebanon; ^3^Neuropediatrics, Hopital Universitaire des Enfants Reine Fabiola (HUDERF), Brussels, Belgium; ^4^Inserm U 1127, CNRS UMR 7225, Sorbonne Universités, UPMC Univ Paris 06 UMR S 1127, Institut du Cerveau et de la Moelle épinière (ICM), F-75013 Paris, France; ^5^AP-HP, Groupe Hospitalier Pitié-Salpêtrière, Département de Génétique, 75013 Paris, France; ^6^Service de Pediatrie, Hotel-Dieu de France, Beirut, Lebanon; ^7^Institut Jérôme Lejeune, Paris, France; ^8^Neuropediatrics Department, Rafic Hariri University Hospital, Beirut, Lebanon; ^9^Institute of Human Genetics, University Hospital Essen, University Duisburg-Essen, Essen, Germany; ^10^INOVIE, Beirut, Lebanon

## Abstract

Dravet syndrome, also known as severe myoclonic epilepsy in infancy, is a rare disease characterized by the appearance of different types of seizures in a healthy baby, triggered by various factors and stressful events. We report 8 Lebanese cases referred for molecular analysis of the *SCN1A* gene. Results were positive in 7 cases and revealed de novo variants at the heterozygous state in different exons of the gene for all except one, where the variant was intronic. Four variants were novel. Confirmation of Dravet syndrome is important for a better follow-up and treatment, preventing the occurrence of status epilepticus and severe neurological deterioration.

## 1. Introduction

Dravet syndrome (DS), also known as severe myoclonic epilepsy in infancy (SMEI), is an autosomal dominant genetic disorder, initially described in 1978, by Charlotte Dravet [1]. It is characterized by seizures of any type, usually prolonged hemi-clonic or generalized tonic-clonic, appearing within the first year of life, febrile in the beginning then turning to afebrile, and resistant to most of the anti-epileptic drugs that generally have efficient results in other types of seizure. Afterwards, a slowing of the psychomotor development, neurological and cognitive deficits, and the appearance of behavioral disturbances are noted. Neuroimaging studies show nonspecific findings, and diagnosis is given based on the clinical state of the patient in addition to the genetic testing [2].

Around 80% of affected patients with DS carry a mutation in the alpha-1 subunit of the sodium channel of the *SCN1A* gene localized in 2q24.3, encoding a voltage-gated sodium channel essential for the excitability of neurons. The vast majority of these variants are de novo [3]. In 5–10%, the variants are inherited (typically missense mutations), and the considered diagnosis is thus part of the genetic epilepsy with febrile seizures-plus (GEFS+) spectrum. Somatic mosaic mutations have also been reported [4]. A minority of DS patients might have pathogenic variants in other genes such as *PCDH19*, *SCN1B*, *SCN8A*, *HCN1*, *GABRA1*, and *GABRG2* [5], and other pathologies close to DS might then be considered as differential diagnosis. Mutations in the *PCDH19* gene (Xq22.1) are thought to account for about 5% of female DS cases [5].

Here, we report a study on gene sequencing of the *SCN1A* gene in eight Lebanese cases with Dravet syndrome. We will highlight and focus the symptoms, the mutations of the diagnosed patients, and the treatment to be given.

## 2. Materials and Methods

### 2.1. Case Study

From January 1999 till December 2016, eight unrelated Lebanese children aged between 6 and 18 months were referred for genetic counselling and molecular analysis of the *SCN1A* gene because of suspicion of DS. Informed written consent was obtained from the patients' parents before blood sampling.

### 2.2. Molecular Analysis

Genetic testing was performed on peripheral blood samples, by sequencing the coding regions of *SCN1A* bidirectionally and using MLPA (Multiplex Ligation dependent Probe Amplification), as previously described by Depienne et al. [5].

Parents were tested by Sanger analysis for the specific mutation found in their affected children.

## 3. Results

The 8 children were referred after the age of 1 year for molecular confirmation of DS. All cases were sporadic, and none had any family history of seizures or of neurological diseases. They had approximately the same clinical history: they all had normal cognitive and psychomotor development till the age of 5-6 months. At that point, they suffered from prolonged convulsions triggered by fever. Seizures were short in the beginning, lasted for less than 5 minutes, and healed by themselves. With time, they turn to become afebrile, lasted for longer duration (between 10 and 20 minutes), thus requiring the need of acute treatment to stop the convulsions. Seizure types were either generalized, in 5 of the 8 patients, or focal in the case of the 3 others, with a frequency ranging from 1 to 4 seizures per month. The generalized seizures in the 5 patients were associated with tonic-clonic movements of the 4 extremities, perioral cyanosis, uprolling of the eyes, and a short post-ictal phase of less than 30 minutes. Two of the patients with focal seizures had abnormal clonic movements in the upper right extremity, and one had tonic-clonic movements in the right upper and lower extremities with deviation of the mouth during the crisis to the right side. One out of the 8 patients also had myoclonic jerks. The crises were noncured or partially cured by anti-epileptic drugs. In three of the patients, neurocognitive decline was already present.

Later, the molecular results were communicated to the families; 3 patients had lack of follow-up. Two patients amongst those having generalized seizures died before the age of 7 years. All others had persistent seizures despite the administration of many anti-epileptic drugs and were completely dependent on their parents and suffered from a poor quality of life.

**Figure 1:**
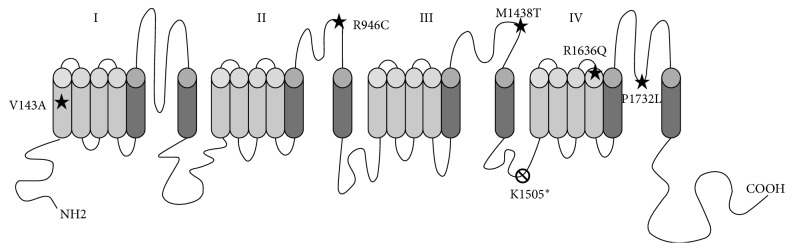
Position on the Nav1.1 channel encoded by the *SCN1A* gene of the mutations found in this study.

Sequencing of *SCN1A* revealed a mutation in 7 patients (87%) localized in different exons and introns (Table 1, Figure 1). All variants were de novo and found at the heterozygous state, and 4 were novel (Table 1). The variants were classified as pathogenic or likely pathogenic. Five were missense variants, one was a nonsense mutation, and one was a variant altering the donor splice site of intron 4.

## 4. Discussion

DS is a rare neurological disease, mainly diagnosed clinically and by genetic testing once it is suspected. Its prevalence worldwide is estimated to be <1/40,000 [9].

In the Arab world, no case series and no statistics are available. Few studies were published presenting case reports of patients diagnosed as having DS. In Tunisia, 4 unrelated families, referred for a suspicion of DS, were studied [10, 11]. Molecular analysis and direct gene sequencing of the *SCN1A* gene showed a known mutation (c.1811G > A) and a putative disease-associated haplotype in only one family. These families were classified subsequently as having genetic epilepsy with febrile seizures plus (GEFS+) [10, 11]. In the United Arab Emirates, 3 male siblings with a possibility of mosaicism in the parents were reported [12]. Two siblings of a nonconsanguineous Palestinian family with confirmed parental mosaicism were reported [13]. Finally, one case was reported in Lebanon in 2016 of a DS patient and immunodeficiency [14].

In Lebanon, this is the first study series on DS. The patients of the present series had the typical signs and symptoms of DS: febrile seizures before the age of 1 year, prolonged seizures resistant to treatment, clinical deterioration with time, and no family history of seizure.

It is noteworthy that for a period of 17 years, only 8 cases were referred for molecular analysis to our center. If we consider that the same number was referred to the other genetic center of the country, we can thus estimate a prevalence of DS of 1/90,000 in Lebanon. This low proportion of cases is most probably due to the fact that DS cases are not diagnosed, or misdiagnosed with any febrile seizure or neurological problem, or because of lack of follow-up and unavailability of genetic tests. In our series, only *SCN1A* was tested. Our proportion of positive cases with the latter gene is 87.5% as in other studies. No phenotype/genotype correlation was noted when we compared our patients and the one from the literature with the same variants. Nowadays, with the next-generation Sequencing approach, we believe that fewer cases will be missed, allowing better follow-up, in order to provide the patient with the appropriate treatment, control resistance of the drugs towards seizures, and slowing the deterioration caused by this disease.

Treatment of DS is complicated. Some medications are useful to decrease the frequency of seizures and the severity of the disease such as stiripentol, valproate, benzodiazepine, and topiramate. Besides medication, controlling infections and body temperature variations also showed to decrease the frequency of seizures and severity of the disease [15], whereas ketogenic diet and vagal stimulation can be used as alternative treatment. On the contrary, some anti-epileptic drugs, such as carbamazepine, oxcarbazepine, lamotrigine, and phenytoin aggravate seizures in DS [15]. It is important to note that many of the patients are undertreated and do not receive the standard care for DS due to incorrect diagnosis. The patients reported herein had their symptoms improve while on valproate, topiramate, levetiracetam, and phenobarbital. These drugs help only in reducing the severity of the seizures, without complete eradication of the disease.

Thus, it is advised to closely follow-up the cases to prevent such hurdles and to have a multidisciplinary specialized follow-up, with pediatricians, nurses, psychologists, physiotherapists, a home care team, and an educational system to patients and their parents.

## Figures and Tables

**Table 1:** Types of *SCN1A* mutations found in the Lebanese patients.

Intron/exon	Variant type	cDNA change	Protein change	Inheritance	Previously described
Exon 3	Missense	c.428T > C	p.(Val143Ala)	*De novo*	No
Intron 4	Duplication	c.602 + 2dupT	p.?	*De novo*	No
Exon 15	Missense	c.2836C > T	p.(Arg946Cys)	*De novo*	Fukuma [6]
Exon 22	Missense	c.4313T > C	p.(Met1438Thr)	*De novo*	No
Exon 24	Nonsense	c.4513A > T	p.(Lys1505^*∗*^)	*De novo*	No
Exon 26	Missense	c.4907G > A	p.(Arg1636Gln)	*De novo*	Harkin et al. [7]
Exon 26	Missense	c.5195C > T	p.(Pro1732Leu)	*De novo*	Bayat et al. [8]
